# Ruptured Bullae: A Case of Transthyretin Cardiac Amyloidosis

**DOI:** 10.7759/cureus.16318

**Published:** 2021-07-11

**Authors:** John Dayco, Martin Weaver, Nabeel Sumbal, Rebecca Theisen, Shaheena Raheem

**Affiliations:** 1 Department of Internal Medicine, Wayne State University Detroit Medical Center, Detroit, USA; 2 Office of Learning and Teaching, Wayne State University School of Medicine, Detroit, USA; 3 College of Engineering, University of Michigan, Ann Arbor, USA; 4 Department of Internal Medicine and Pediatrics, Wayne State University Detroit Medical Center, Detroit, USA

**Keywords:** transthyretin amyloid cardiomyopathy, perfusion mri, echocardiogram, cardiovascular disorders, heart failure

## Abstract

Amyloidosis is a rare disease with an incidence of only 16.6 per 100,000 patients per year. A high grade of clinical suspicion is required to suspect an atypical cause of left ventricular hypertrophy or new-onset heart failure. A transthoracic echocardiogram (TTE) is the initial evaluation that may yield clues pointing towards an etiology of cardiac amyloidosis. Due to the subjective nature of TTE interpretations, suspicion for cardiac amyloidosis may be missed. Once suspicion arises, additional tests, such as serum and urine electrophoresis and technetium-99m pyrophosphate myocardial perfusion imaging, can further aid in establishing a diagnosis. The pathophysiology in transthyretin amyloidosis (ATTR) involves the misfolding of the transthyretin/prealbumin protein, which leads to an inherent propensity to aggregate. These proteins can accumulate in the extracellular space between cardiac myocytes, which may thicken sections of the heart, leading to ventricular restriction. Here, we explore the case of an 83-year-old man with chronic, treatment-resistant heart failure with preserved ejection fraction, New York Heart Association class III, who presented with multiple ruptured bullae in the bilateral lower extremity, leading to a new diagnosis of ATTR cardiac amyloidosis.

## Introduction

Cardiac amyloidosis is an underdiagnosed cause of left ventricular hypertrophy by which misfolded protein aggregates invade the extracellular space of cardiac tissue resulting in hypertrophy and restrictive cardiomyopathy. Amyloidosis is a rare disease with an incidence of 16.6 per 100,000 patients per year, and a prevalence of 55.2 per 100,000 patients per year in 2012 [1]. Several forms of amyloid can deposit in the cardiac extracellular space including amyloid, which is a misfolding of monoclonal immunoglobulin (Ig) light chains often associated with plasma cell dyscrasias. Transthyretin amyloidosis (ATTR) occurs when transthyretin/prealbumin proteins misfold, leading to aggregation within the extracellular space. Also known as senile amyloidosis, ATTR commonly presents in patients after the age of 60 without provocation. Although clinical presentation may vary, classically, it may include carpal tunnel syndrome or spinal stenosis outside of ventricular hypertrophy [2]. While there are no current guideline-directed screening recommendations for amyloidosis, a secondary cause should be investigated for patients who present with unexplained ventricular hypertrophy.

## Case presentation

An 83-year-old man with a history of congestive heart failure, chronic diabetes mellitus, and essential hypertension presented to the emergency department for the third time in three months with complaints of bilateral lower extremity swelling and dyspnea on exertion. His concern for the current visit was new-onset blisters in the bilateral anterior legs that had developed, burst, and had been draining serosanguinous fluid for the last week.

**Figure 1:**
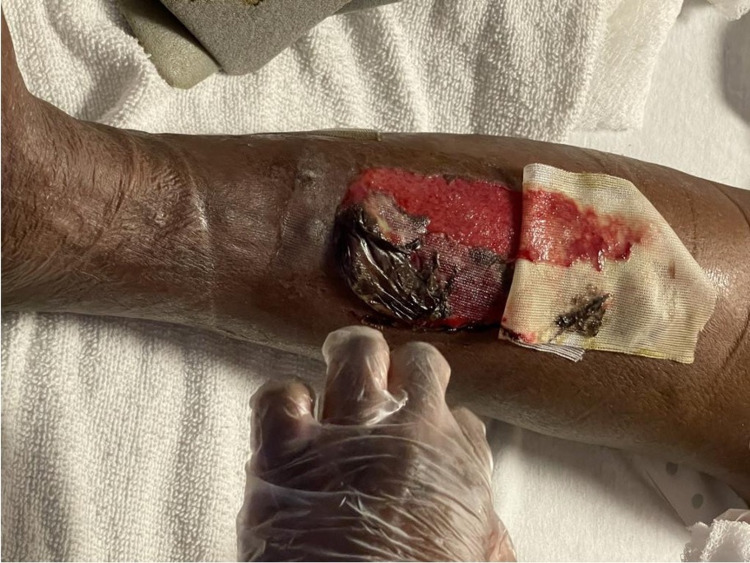
Ruptured bullae (4-6 cm) as seen along the anterior portion of bilateral legs.

Examination of the patient revealed bilateral basilar crackles, diminished breath sounds on the right side, a jugular venous pulse of 8 cm, and severe bilateral 4+ lower extremity edema with skin breakdown and multiple ruptured/deroofed bullae, measuring 4-6 cm, which were draining serous fluid (Figure 1).

**Figure 2:**
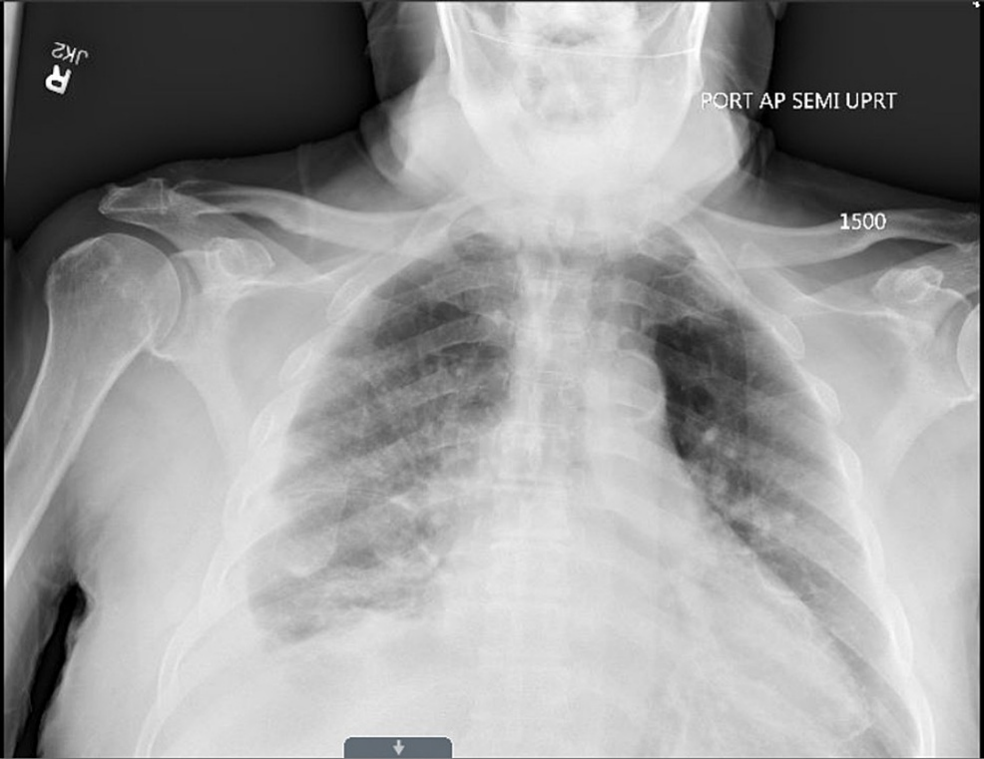
Chest X-ray on admission demonstrates cardiomegaly with pulmonary vascular congestion and a small right-sided pleural effusion.

An electrocardiogram (EKG) showed a low-voltage normal sinus rhythm. The patient’s brain natriuretic peptide was elevated to 450 pg/mL. His troponin was also elevated at 94 ng/L, which has been around the same for the last 12 months. A chest X-ray (Figure 2) demonstrated cardiomegaly, vascular congestion, and a right-sided pleural effusion. His wound cultures were negative.

**Figure 3:**
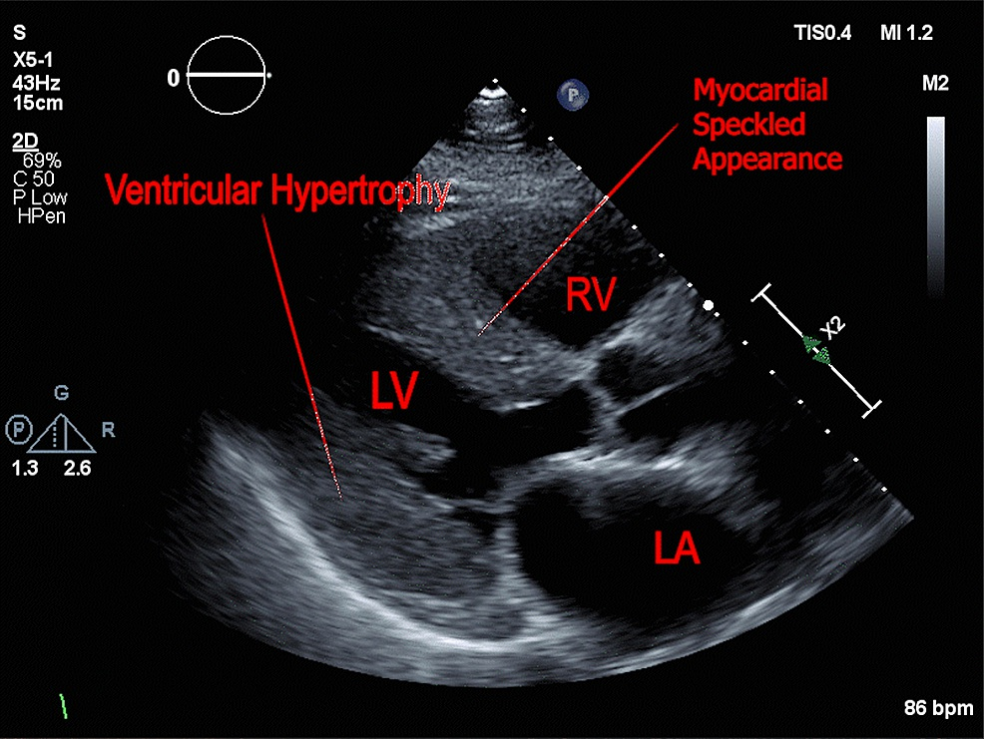
Echocardiogram shows ventricular thickening and myocardial speckled appearance in the interventricular septum.

A transthoracic echocardiogram (TTE) was obtained and demonstrated normal ventricular size and systolic function with an ejection fraction of 55%. In addition, there were findings of a severely increased left ventricular wall thickness with a granular speckled appearance of the myocardium suspicious of cardiac amyloidosis (Figure 3).

**Figure 4:**
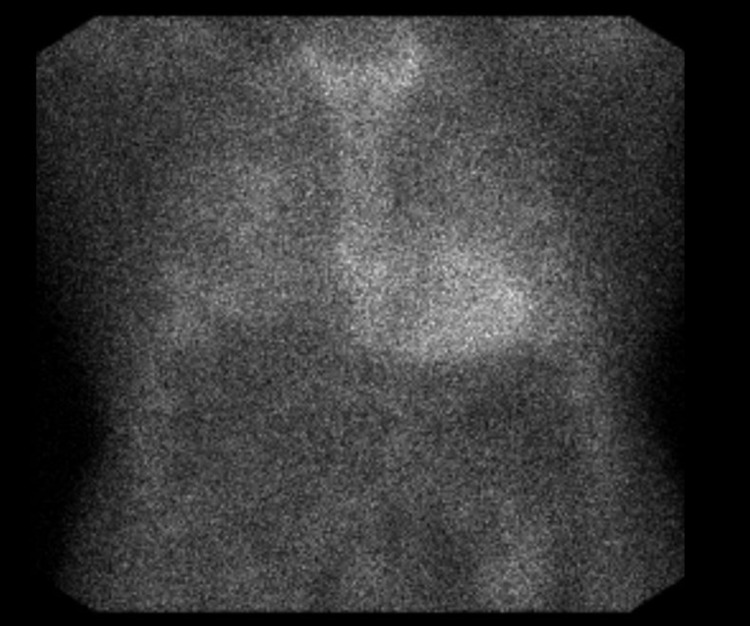
Technetium-99m pyrophosphate myocardial perfusion imaging study demonstrates increased technetium-99m radiotracer uptake of the myocardium, suggestive of transthyretin amyloid cardiomyopathy.

Urine and serum protein electrophoresis was performed which showed mild hypergammaglobulinemia (2.04 g/dL with normal high of 1.98 g/dL) but no bands in urine. Technetium-99m pyrophosphate myocardial perfusion was subsequently performed which showed increased radiotracer uptake of the myocardium compared to the bone with significant retention, suggestive of transthyretin amyloid cardiomyopathy (Figure 4). A saliva sample was collected and sent for transthyretin genetic lab testing.

The patient received aggressive diuresis with intravenous bumetanide, spironolactone, and metolazone. He was discharged on this regimen, with a cardiology outpatient follow-up. The patient was also educated on the importance of adhering to fluid restrictions, a low salt diet, and medication. During his cardiology follow-up visit, the patient had clinically improved. His bilateral pitting edema had reduced to 1+, and the pulmonary auscultation was nonremarkable. The ruptured bullae throughout his anterior legs had healed. The transthyretin genetic testing was pending at the time of writing this report with results to be discussed during his future cardiology follow-up.

## Discussion

ATTR cardiac amyloidosis is a rare, underdiagnosed, and potentially life-threatening type of amyloidosis that may lead to restrictive congestive heart failure. Timely diagnosis is critical in improving patient morbidity and mortality as it enables early intervention. Awareness among physicians remains low, which results in ATTR amyloidosis remaining underdiagnosed, especially in patients with congestive heart failure. This may be because symptoms of ATTR cardiac amyloidosis are similar to other more common causes of heart failure such as hypertension. Physicians may evaluate for signs and symptoms of multiorgan dysfunction, as well as reevaluate their diagnosis for patients who demonstrate suboptimal response to their treatments. Because amyloidosis is a systemic disease, physicians could consider certain clinical findings to help elicit suspicion [3]. Polyneuropathy is a common symptom among amyloidosis patients. In our case, the patient had been treated with gabapentin for many years, which was previously attributed to his diabetes mellitus. Additional clinical findings may also aid in the diagnosis, such as the presence of carpal tunnel syndrome. Certain investigative studies may aid in the diagnosis. For ATTR cardiac amyloidosis, patients often have a low-voltage EKG, especially as they often show ventricular thickening on imaging studies (Figure 2). Another useful laboratory finding is a persistently mildly elevated troponin on repeated lab draws. Our patient had a troponin level of 70-90 ng/L over the last 12 months. Lastly, TTEs may provide a very strong clinical clue for diagnosis. TTE findings may include increased thickness in the atrioventricular valves, interatrial septum, and right ventricular free wall [4]. Additionally, a very strong sign on an echocardiogram is the presence of a speckled appearance in the myocardium (Figure 2). Due to the inherent subjectivity of echocardiogram interpretations, a strong clinical correlation and inter-specialty collaboration are essential in the assessment. Early diagnosis provides tremendous benefits for the patient [3]. Treatments administered in the initial phase of the disease can prevent further deposition of amyloid proteins, which may slow down the disease progression. Current treatment options are also more effective during the early course of the disease and become limited with disease progression. Pharmacological approaches work by stabilizing tetramers, which prevents the formation of fibrils, or by decreasing ATTR production [5].

## Conclusions

Due to the age and progression of our patient’s disease, he was determined to not be a candidate for pharmacological intervention. Had the disease been diagnosed earlier, specific treatments for cardiac amyloidosis may have eased the disease progression. It is crucial for physicians to remain vigilant in the evaluation of uncontrolled congestive heart failure, and to treat not only the symptoms but to determine the etiology of the disease, such as ATTR cardiac amyloidosis.
